# Clinical and demographic factors associated with pediatric difficult intravenous access in the operating room

**DOI:** 10.1111/pan.14438

**Published:** 2022-03-23

**Authors:** Heather A. Ballard, John Hajduk, Eric C. Cheon, Michael R. King, Jeffrey H. Barsuk

**Affiliations:** ^1^ Department of Pediatric Anesthesiology Ann & Robert H. Lurie Children's Hospital of Chicago Chicago Illinois USA; ^2^ Department of Medicine Northwestern University Feinberg School of Medicine Chicago Illinois USA

**Keywords:** catheterization, catheters, child, healthcare disparities, institutional practice, interventional, logistic models, peripheral, retrospective studies, risk factors, ultrasonography

## Abstract

**Background:**

Pediatric intravenous catheter insertion can be difficult in the operating room due to the technical challenges of small diameter vessels and the need to rapidly gain intravenous access in anesthetized children. Few studies have examined factors associated with difficult vascular access in the operating room, especially accounting for the increased possibility to use ultrasound guidance.

**Aims:**

The primary aim of the study was to identify factors associated with pediatric difficult vascular access in the operating room. Our primary hypothesis was that Black race, Hispanic ethnicity, and ultrasound use would be associated with pediatric difficult vascular access.

**Methods:**

We performed a retrospective analysis of prospectively collected data from a cohort of pediatric patients who had intravenous catheters inserted in the operating room at an academic tertiary care children's hospital from March 2020 to February 2021. We measured associations among patients who were labeled as having difficult vascular access (>2 attempts at access) with demographic, clinical, and hospital factors.

**Results:**

12 728 intravenous catheter insertions were analyzed. Multivariable analysis showed significantly higher odds of difficult vascular access with Black non‐Hispanic race (1.43, 95% CI: 1.06–1.93, *p* = .018), younger age (0.93, 95% CI: 0.89–0.98, *p* = .005), overweight (1.41, 95% CI: 1.04–1.90, *p* = .025) and obese body mass index (1.56, 95% 95% CI: 1.12–2.17, *p* = .008), and American Society of Anesthesiologists physical status III (1.54, 95% CI:1.11–2.13, *p* = .01). The attending anesthesiologist compared to all other practitioners (certified registered nurse anesthetist: (0.41, 95% CI: 0.31–0.56, *p* < .001, registered nurse: 0.25, 95% CI: 0.13–0.48, *p* < .001, trainee: 0.21, 95% CI: 0.17–0.28, *p*‐value <.001 with attending as reference variable) and ultrasound use (2.61, 95% CI: 1.85–3.69, *p* < .001) were associated with successful intravenous catheter placement.

**Conclusions:**

Black non‐Hispanic race/ethnicity, younger age, obese/overweight body mass index, American Society of Anesthesiologists physical status III, and ultrasound were all associated with pediatric difficult vascular access in the operating room.

**What is already known**

Previously reported risk factors for difficult vascular access in children include younger age, female sex, darker skin color, obesity, prematurity, end stage renal disease, congenital heart disease, higher American Society of Anesthesiologists physical status, and emergency surgery, but older studies have not accounted for the use of ultrasound and its effects on difficult vascular access in the operating room.

**What this article adds**

Black non‐Hispanic race, younger age, overweight and obese body mass index, American Society of Anesthesiologists physical status III, and ultrasound were associated with pediatric difficult vascular access in the operating room. In patients with difficult intravenous access, the attending anesthesiologist inserting the intravenous catheter was more likely to be successful.

## 1 INTRODUCTION

Intravenous (IV) access is required to safely care for the majority of children undergoing the estimated 6 million pediatric surgical procedures each year in the United States.^1^ Intravenous catheter insertion can be challenging in children due to their smaller caliber blood vessels, with reported first attempt success rates between 39–73%.^2^, ^3^, ^4^ Children that require more than 2 attempts at IV access are labeled as difficult venous access (DVA).^5^

Most of the literature on children with DVA has originated from the care provided in the Emergency Department (ED)^5^, ^6^ with few studies on children undergoing procedures in the operating room (OR).^2^, ^7^, ^8^, ^9^, ^10^ In the OR environment, most children are fasting but are also often anesthetized with anesthetic gases before IV catheter placement, potentially improving insertion through immobilization and venodilation. But this also results in a critical patient safety period where children are prone to hypotension and airway obstruction without IV access. Children with DVA receive delayed care and decreased parental satisfaction with their child's care team.^5^, ^11^

Previously reported risk factors for DVA in children include age less than 1 year old, female sex, darker skin color, Black race, obesity, prematurity, end stage renal disease, congenital heart disease, higher American Society of Anesthesiology (ASA) physical status, and emergency surgery.^2^, ^7^, ^8^, ^9^, ^10^, ^12^, ^13^, ^14^ However, these studies have not accounted for the use of ultrasound in obtaining vascular access and its association with DVA. Ultrasound‐guided intravenous catheter insertion (USGIV) is a relatively new technique that has been shown to increase success rates and decrease time to cannulation in pediatric patients with DVA.^15^, ^16^, ^17^ The use of ultrasound may be helpful in patients with nonvisible veins due to the DVA risk factors of obesity or dark skin color; early application of USGIV might lead to reduction of DVA in these patients.^18^, ^19^

The primary aim of the study was to identify factors associated with pediatric DVA in the OR. Our primary hypothesis was that Black race, Hispanic ethnicity, and ultrasound use would be associated with pediatric DVA because Black and Hispanic patients could have darker skin tones making vein visualization more difficult or because previous research has shown healthcare disparities in these groups.^9^, ^20^, ^21^, ^22^ Ultrasound was expected to be associated with DVA because it is often used as a rescue method in our institution after the surface landmark technique has failed.

## 2 METHODS

### 2.1 Study design & setting

We conducted a retrospective analysis of prospectively collected data from a cohort of all pediatric patients who had an IV catheter inserted in the OR at the Ann and Robert H. Lurie Children's Hospital of Chicago, a tertiary care, urban pediatric hospital between March 2020 and February 2021. We evaluated demographic, comorbidity, and hospital characteristics of patients to determine whether they were associated with pediatric DVA. We followed the Strengthening the Reporting of Observational Studies in Epidemiology (STROBE) guidelines (www.strobe‐statement.org) while performing and reporting this study. We obtained study approval from the Lurie's Children's Hospital Institutional Review Board (IRB 2019–2860, approved 6/10/19, chairperson Jennifer Rubin), and the requirement for written informed consent was waived by the IRB. IRB approval was initially obtained for another prospective study evaluating IV insertion outcomes after an educational intervention.^23^

### 2.2 Procedure

All patients who underwent IV insertion in the OR were evaluated through a query searching for an IV catheter insertion note in the electronic medical record (EMR), Epic (Verona, WI). Information was extracted from the note about the number of IV attempts, practitioner placing the IV, and use of ultrasound. At the study institution, it is routine practice to document this information for every IV inserted in the operating room. The EMR was also queried for each patients' demographic variables [self‐reported race/ethnicity, age, sex, weight, and body mass index (BMI)], comorbidities (presence of renal disease, cardiac disease, prematurity, and ASA status), and hospital variables (practitioner type inserting the IV, ultrasound use, inpatient vs. outpatient status, emergency vs. elective surgery, and surgical subspecialty performing the operation).

### 2.3 Data sources/measurement

We defined difficult vascular access as more than 2 attempts at peripheral IV catheter insertion as has been done in prior studies.^3^, ^6^, ^7^, ^16^ Race and ethnicity were modeled as White non‐Hispanic, Black non‐Hispanic, or White Hispanic. Other racial and ethnic groups were excluded from the analysis due to the small numbers (<5%) of each of these groups.

BMI percentiles were measured using the Centers of Disease Control and Prevention BMI percentile calculator.^24^ After converting these BMI percentiles, the values were separated into four categories: underweight (<5th percentile), healthy weight (5 to <85th percentile, overweight (85th to <95th percentile), and obese (>95th percentile).^25^ As there are no established norms for BMI in patients who are less than 2 years old, BMI percentiles could not be calculated for these patients.

Comorbidities were abstracted from the International Classification of Disease 9 and 10 Clinical Modification (ICD‐9‐CM, ICD‐10‐CM) codes associated with each patient's medical record. ICD‐9‐CM and ICD‐10‐CM codes were defined as follows: renal disease (580–589, 753, N00‐08, P96, Q61–64, N10–16, N17–19), congenital heart disease (745–747, Q20–28), and prematurity (765, P05, P07).

#### 2.3.1 Statistical methods

Chi‐squared tests (categorical data) and Wilcoxon Rank Sum tests (continuous variables) were used to compare DVA with demographic, comorbid, and hospital variables. Both univariable and multivariable logistical regression model was used to model the relationship between difficult vascular access and demographic factors (race/ethnicity, age, sex, weight, and BMI category), patient comorbidities (history of renal disease, cardiac disease, or prematurity, ASA status), and hospital factors (practitioner type, ultrasound use, patient class, emergency status, and surgical subspecialty). Based upon the reviewer's suggestion, we performed an additional analysis to model the relationship between ultrasound use and the demographic factors, patient comorbidities, and hospital factors above. The logistic regression model was estimated with standard errors adjusted for clustering of insertions for the same patient. All statistical analysis was conducted using Stata Version 15.0 (College Station, TX).

## 3 RESULTS

**Figure 1:**
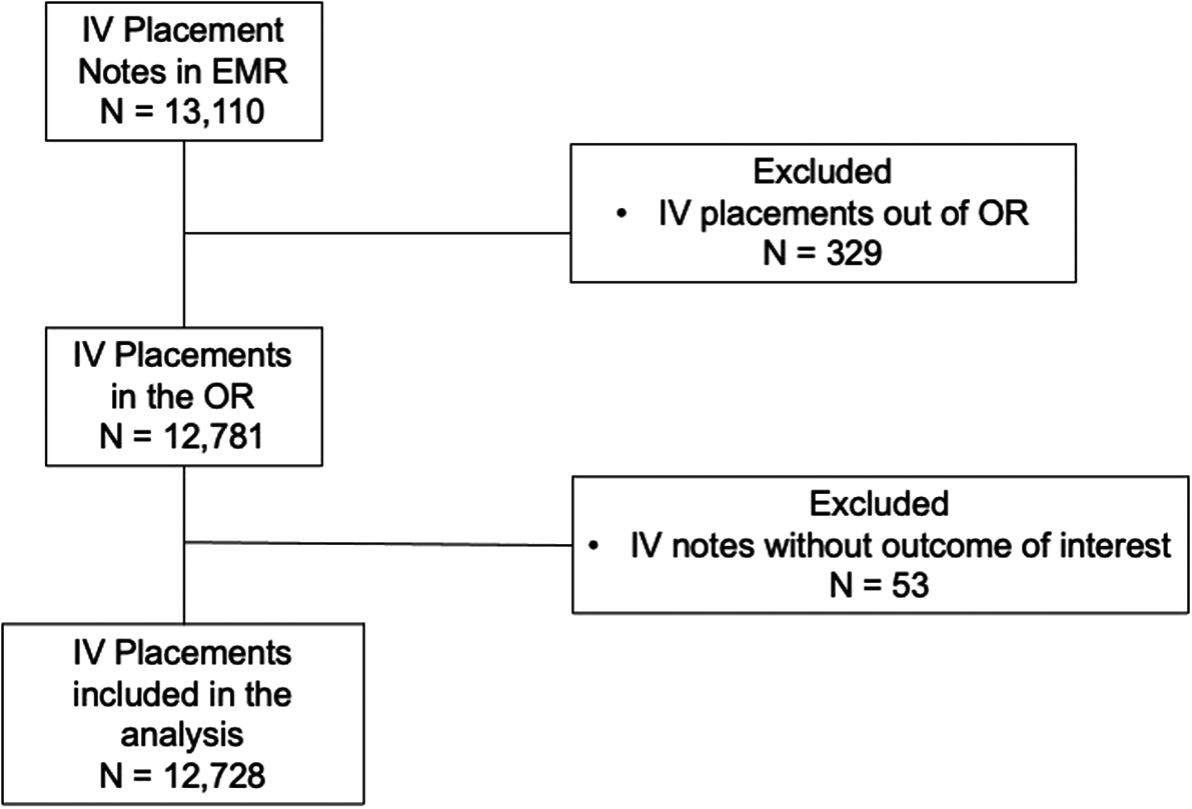
Inclusion and exclusion criteria flow diagram

A total of 13 110 IV catheter insertions were completed by anesthesia practitioners during the study period. Three hundred twenty‐nine insertions were excluded due to IV insertion outside of the OR (*n* = 329) or due to missing data on number of IV attempts (*n* = 53) in the procedure note, for a total of 12 728 IV placements included in the analysis (Figure 1). The first attempt success rate was 78.0%. Characteristics of patients with and without DVA are listed in Table 1. Eight hundred sixty‐six (7.3%) patient IV insertions were identified as DVA. In the multivariable analysis, factors associated with DVA included the following: racial/ethnic group (*p* = .009), younger age (*p* < .001), male sex (*p* = .045), lower weight (*p* < .001), higher BMI category (*p* < .001), presence of renal disease (*p* = .030), ASA III compared to ASA I status (*p* = .026), practitioner type inserting the IV successfully (*p* < .001), use of ultrasound (*p* < .001), nonemergency status (*p* = .033), and surgical subspecialty (*p* < .001).

**Table 1:** Baseline demographic, comorbidities, and hospital characteristics of patients without and with Difficult Vascular Access^a^

	Nondifficult vascular access (*N* = 11 862)	Difficult vascular access (*N* = 866)	*p*‐value
Race			
White non‐Hispanic	5652 (47.7)	389 (44.9)	.009
White Hispanic	3258 (27.5)	216 (24.9)	
Black non‐Hispanic	1430 (12.1)	135 (15.6)	
Age, months (median, IQR)	80 (32–160)	30 (10–84)	<.001
Sex			
Female	5127 (43.2)	337 (38.9)	.045
Male	6734 (56.8)	529 (61.1)	
Weight, kg (median, IQR)	23.0 (13.2–50.0)	12.8 (8.4–27.1)	<.001
Body mass index category	62.1 (24.6–88.9)	73.8 (31.2–94.5)	<.001
Underweight (<5 percentile)	846 (9.8)	44 (9.9)	
Healthy weight (5–84th percentile)	5206 (60.3)	222 (50.0)	
Overweight (85–94th percentile)	1171 (13.6)	73 (16.4)	
Obese (95th percentile or greater)	1410 (16.3)	105 (23.7)	
Renal disease	786 (6.6)	74 (8.6)	.030
Cardiac disease	1197 (10.1)	97 (11.2)	.297
Prematurity	115 (1.0)	14 (1.6)	.066
ASA physical status			
I	3604 (30.4)	244 (28.2)	.026
II	4700 (39.6)	317 (36.6)	
III	3144 (26.5)	272 (31.4)	
IV	409 (3.5)	33 (3.8)	
Practitioner type			
Attending anesthesiologist	3943 (34.0)	576 (67.7)	<.001
CRNA	1779 (15.4)	92 (10.8)	
RN	551 (4.8)	14 (1.7)	
Trainee	5309 (45.8)	169 (19.9)	
Ultrasound used	1101 (9.4)	181 (21.3)	<.001
Patient class			
Outpatient	9549 (80.5)	716 (82.7)	.124
Inpatient	2307 (19.5)	150 (17.3)	
Emergency status	111 (0.9)	2 (0.2)	.033
Surgical subspecialty			
Otolaryngology	1980 (16.7)	157 (18.1)	<.001
Cardiology	508 (4.3)	25 (2.9)	
Cardiac surgery	414 (3.5)	13 (1.5)	
Dentistry	571 (4.8)	25 (2.9)	
Dermatology	230 (1.9)	5 (0.6)	
Gastroenterology	906 (7.6)	39 (4.5)	
Medical imaging	1314 (11.1)	139 (16.1)	
Neurosurgery	458 (3.9)	34 (3.9)	
Ophthalmology	493 (4.2)	40 (4.6)	
Orthopedics	1137 (9.6)	51 (5.9)	
Pediatric surgery	1653 (13.9)	141 (16.3)	
Plastics	515 (4.3)	35 (4.0)	
Urology	1382 (11.7)	140 (16.2)	

Abbreviations: ASA, American Society of Anesthesiologists; CRNA, certified registered nurse anesthetist; IQR, interquartile range; RN, registered nurse.

^a^ Body mass index data missing for all children less than 2 years old (28%) because there are no norms of children in this age group.

The results of the univariable logistic regression are shown in Table 2. The demographic variables significantly associated with DVA included the following: Black non‐Hispanic (OR: 1.37, 95% CI: 1.12–1.68, *p* = .002), younger age (OR: 0.91, 95% CI: 0.89–0.92, *p* < .001), male sex (OR: 1.19, 95% CI: 1.04–1.38, *p* = .014), lower weight (OR: 0.98, 95% CI: 0.98–0.99, *p* < .001), overweight BMI (OR: 1.46, 95% CI: 1.11–1.92, *p* = .006), and obese BMI (OR: 1.75, 95% CI: 1.37–2.22, *p* < .001). The patient comorbidities associated with DVA were renal disease (OR: 1.32, 95% CI: 1.03–1.69, *p* = .030) and ASA class III (OR: 1.28, 95% CI: 1.07–1.53, *p* = .007). Presence of cardiac disease and prematurity were not significantly associated with DVA. Hospital variables significantly associated with DVA were insertion by the attending anesthesiologist compared to all other categories of practitioners (certified registered nurse anesthetist [CRNA]: OR: 0.35, 95% CI: 0.28–0.44, *p* < .001), registered nurse [RN]: OR: 0.17, 95% CI: 0.10–0.30, *p* < .001, trainee: OR: 0.22, 95% CI: 0.18–0.26, *p* < .001 with attending as reference variable) and the use of an ultrasound for IV insertion (OR: 2.62, 95% CI: 2.20–3.13, *p* < .001). When compared to the reference category of otolaryngology, cardiology, cardiac surgery, dentistry, dermatology, gastroenterology, and orthopedics were significantly less likely to be associated with pediatric DVA. Medical imaging and urological procedures were significantly more associated to be associated with pediatric DVA.

**Table 2:** Univariable Logistic Regression to Evaluate Association of Demographic, Comorbidities, and Hospital Characteristics of Patients with Difficult Vascular Access^a^

	Odds ratio	95% CI	*p*‐value
Race			
White non‐Hispanic	Reference		
White Hispanic	0.96	0.81–1.14	.670
Black non‐Hispanic	1.37	1.12–1.68	.002
Age (year)	0.91	0.89–0.92	<.001
Sex			
Female	Reference		
Male	1.19	1.04–1.38	.014
Weight (kg)	0.98	0.98–0.99	<.001
Body mass index category			
Underweight (<5 percentile)	Reference		
Healthy weight (5–84th percentile)	1.22	0.88–1.70	.240
Overweight (85–94th percentile)	1.46	1.11–1.92	.006
Obese (95th percentile or greater)	1.75	1.37–2.22	<.001
Renal disease	1.32	1.03–1.69	.030
Cardiac disease	1.12	0.90–1.40	.297
Prematurity	1.68	0.96–2.94	.069
ASA physical status			
I	Reference		
II	1.00	0.84–1.18	.966
III	1.28	1.07–1.53	.007
IV	1.19	0.82–1.74	.363
Practitioner type			
Attending anesthesiologist	Reference		
CRNA	0.35	0.28–0.44	<.001
RN	0.17	0.10–0.30	<.001
Trainee	0.22	0.18–0.26	<.001
Ultrasound used	2.62	2.20–3.13	<.001
Patient class			
Outpatient	Reference		
Inpatient	0.87	0.72–1.04	.124
Emergency status	0.25	0.060–0.99	.049
Surgical subspecialty			
Otolaryngology	Reference		
Cardiology	0.62	0.40–0.96	.031
Cardiac surgery	0.40	0.22–0.70	.002
Dentistry	0.55	0.36–0.85	.007
Dermatology	0.27	0.11–0.67	.005
Gastroenterology	0.54	0.38–0.78	.001
Medical imaging	1.33	1.05–1.69	.018
Neurosurgery	0.94	0.64–1.38	.737
Ophthalmology	1.02	0.71–1.47	.901
Orthopedics	0.57	0.41–0.78	.001
Pediatric surgery	1.08	0.85–1.36	.545
Plastics	0.89	0.59–1.25	.425
Urology	1.28	1.01–1.62	.044

Abbreviations: ASA, American Society of Anesthesiologists; CI, confidence interval; CRNA, certified registered nurse anesthetist; RN: registered nurse.

^a^ Body mass index data missing for all children less than 2 years old (28%) because there are no norms of children in this age group.

The results of the multivariable logistic regression are shown in Table 3. In the multivariate analysis, demographic variables significantly associated with DVA included: Black non‐Hispanic (OR: 1.43, 95% CI: 1.06–1.93, *p* = .018), younger age (OR:0.93, 95% CI: 0.89–0.98, *p* = .005), overweight BMI (OR: 1.41, 95% CI: 1.04–1.90, *p* = .025), and obese BMI (OR: 1.56, CI: 1.12–2.16, *p* = .008). The only patient comorbidity associated with DVA was ASA III class (OR: 1.54, 95% CI: 1.11–2.13, *p* = .010). Presence of cardiac disease, renal disease, and prematurity were not significantly associated with DVA. Hospital variables significantly associated with DVA were insertion by the attending anesthesiologist compared to all other categories of practitioners (certified registered nurse anesthetist [CRNA]: OR: 0.41, 95% CI: 0.31–0.56, *p* < .001), registered nurse [RN]: OR: 0.25, 95% CI: 0.13–0.48, *p* < .001, trainee: OR: 0.21, 95% CI: 0.17–0.28, *p* < .001 with attending as reference variable) and the use of an ultrasound for IV insertion (OR: 2.61, 95% CI: 1.85–3.69, *p* < .001). When compared to the reference category of otolaryngology, cardiology (OR: 0.36, 95% CI: 0.19–0.67, *p* = .001), cardiac surgery (OR: 0.21, 95% CI: 0.07–0.63, *p* = .005), and dermatology (OR: 0.23, 95% CI: 0.05–0.94, *p* = .041) were significantly less likely to be associated with pediatric DVA.

**Table 3:** Multivariable Logistic Regression to Evaluate Association of Demographic, Comorbidities, and Hospital Characteristics of Patients with Difficult Vascular Access^a^

	Odds ratio	95% CI	*p*‐value
Race			
White non‐Hispanic	Reference		
White Hispanic	1.00	0.78–1.29	.986
Black non‐Hispanic	1.43	1.06–1.93	.018
Age (year)	0.93	0.89–0.98	.005
Sex			
Female	Reference		
Male	1.07	0.87–1.32	.504
Weight (kg)	1.00	0.99–1.01	.838
Body mass index category			
Underweight (<5 percentile)	Reference		
Healthy weight (5–84th percentile)	1.20	0.84–1.71	.309
Overweight (85–94th percentile)	1.41	1.04–1.90	.025
Obese (95th percentile or greater)	1.56	1.12–2.17	.008
Renal disease	1.46	0.97–2.19	.070
Cardiac disease	0.75	0.47–1.19	.217
Prematurity	1.25	0.32–4.85	.742
ASA physical status			
I	Reference		
II	1.30	0.98–1.72	.071
III	1.54	1.11–2.13	.010
IV	1.01	0.40–2.51	.988
Practitioner type			
Attending anesthesiologist	Reference		
CRNA	0.41	0.31–0.56	<.001
RN	0.25	0.13–0.48	<.001
Trainee	0.21	0.17–0.28	<.001
Ultrasound used	2.61	1.85–3.69	<.001
Patient class			
Outpatient	Reference		
Inpatient	0.88	0.62–1.24	.456
Emergency status	0.34	0.044–2.58	.296
Surgical subspecialty			
Otolaryngology	Reference		
Cardiology	0.36	0.19–0.67	.001
Cardiac surgery	0.21	0.07–0.63	.005
Dentistry	0.69	0.38–1.24	.214
Dermatology	0.23	0.05–0.94	.041
Gastroenterology	0.81	0.53–1.26	.354
Medical imaging	0.96	0.68–1.34	.794
Neurosurgery	0.49	0.21–1.14	.097
Ophthalmology	1.1	0.59–1.90	.841
Orthopedics	0.86	0.56–1.32	.484
Pediatric surgery	1.04	0.74–1.46	.823
Plastics	0.43	0.18–1.00	.051
Urology	0.99	0.65–1.48	.937

Abbreviations: ASA, American Society of Anesthesiologists; CI, confidence interval; CRNA, certified registered nurse anesthetist; RN, registered nurse.

^a^ Body mass index data missing for all children less than 2 years old (28%) because there are no norms of children in this age group.

The results of the multivariable logistic regression for ultrasound use are shown in Table 4. In the multivariable analysis, demographic variables significantly associated with ultrasound use included: Black non‐Hispanic race (OR: 1.50, 95% CI: 1.15–1.96, *p* = .003), younger age (OR: 0.93, 95% CI: 0.90–0.97, *p* < .001), female sex (OR: 0.68, 95% CI: 0.56–0.81, *p* < .001), higher weight (OR: 1.02, 95% CI: 1.01–1.02, *p* < .001), overweight BMI (OR: 1.54, 95% CI: 1.18–2.01, *p* < .001), and obese BMI (OR: 1.65, CI: 1.22–2.23, *p* < .001). The patient comorbidities associated with ultrasound use were cardiac disease (OR: 1.81, CI: 1.36–2.41, *p* < .001), ASA II (OR: 1.92, 95% CI: 1.33–2.77, *p* = .001), ASA III (OR: 5.58, 95% CI: 3.86–8.07, *p* < .001), and ASA IV class (OR: 10.47, 95% CI: 6.19–17.70, *p* < .001). Presence of renal disease and prematurity were not significantly associated with ultrasound use. Hospital variables significantly associated with ultrasound use were insertion by the attending anesthesiologist compared to other categories of practitioners (registered nurse [RN]: OR: 0.05, 95% CI: 0.014–0.17, *p* < .001, trainee: OR: 0.62, 95% CI: 0.51–0.76, *p* < .001 with attending as reference variable). When compared to the reference category of otolaryngology, cardiology (OR: 6.16, 95% CI: 4.11–9.25, *p* < 0.001), cardiac surgery (OR: 2.16, 95% CI: 1.30–3.58, *p* = .003), medical imaging (OR: 1.90, 95% CI: 1.33–2.73, *p* < .001), orthopedic surgery (OR: 2.65, 95% CI: 1.83–3.85, *p* < .001), pediatric surgery (OR: 1.97, 95% CI: 1.38–2.82, *p* < .001), and plastic surgery (OR: 2.03, 95% CI: 1.07–3.86, *p* = .031) were significantly more likely to be associated with ultrasound use.

**Table 4:** Multivariable Logistic Regression to Evaluate Association of Demographic, Comorbidities, and Hospital Characteristics of Patients with Ultrasound Use^a^

	Ultrasound use (*N* = 1282)	Non‐ultrasound use (*N* = 11 446)	*p*‐value
	Odds Ratio	95% CI	
Race			
White non‐Hispanic	Reference		
White Hispanic	1.02	0.82–1.27	.863
Black non‐Hispanic	1.50	1.15–1.96	.003
Age (year)	0.93	0.90–0.97	<.001
Sex			
Female	Reference		
Male	0.68	0.56–0.81	<.001
Weight (kg)	1.02	1.01–1.02	<.001
Body mass index category			
Underweight (<5 percentile)	Reference		
Healthy weight (5–84th percentile)	1.15	0.83–1.59	.404
Overweight (85–94th percentile)	1.54	1.18–2.01	.001
Obese (95th percentile or greater)	1.65	1.22–2.23	.001
Renal disease	1.27	0.93–1.73	.133
Cardiac disease	1.81	1.36–2.41	<.001
Prematurity	1.21	0.42–3.44	.725
ASA physical status			
I	Reference		
II	1.92	1.33–2.77	.001
III	5.58	3.86–8.07	<.001
IV	10.47	6.19–17.70	<.001
Practitioner type			
Attending anesthesiologist	Reference		
CRNA	0.90	0.70–1.16	.408
RN	0.05	0.014–0.17	<.001
Trainee	0.62	0.51–0.76	<.001
Patient class			
Outpatient	Reference		
Inpatient	3.48	2.75–4.40	<.001
Emergency status	1.50	0.81–2.77	.196
Surgical subspecialty			
Otolaryngology	Reference		
Cardiology	6.16	4.11–9.25	<.001
Cardiac surgery	2.16	1.30–3.58	.003
Dentistry	1.76	0.94–3.32	.079
Gastroenterology	1.17	0.69–1.97	.567
Medical imaging	1.90	1.33–2.73	<.001
Neurosurgery	1.08	0.65–1.78	.775
Ophthalmology	0.48	0.15–1.57	.224
Orthopedics	2.65	1.83–3.85	<.001
Pediatric surgery	1.97	1.38–2.82	<.001
Plastics	2.03	1.07–3.86	.031
Urology	1.27	0.77–2.08	.350

Abbreviations: ASA, American Society of Anesthesiologists; CI, confidence interval; CRNA, certified registered nurse anesthetist; RN, registered nurse.

^a^ Body mass index data missing for all children less than 2 years old (28%) because there are no norms of children in this age group.

## 4 DISCUSSION

Our study found that non‐Hispanic Black race/ethnicity, younger age, higher BMI, higher ASA status, and otolaryngology procedures were associated with higher odds of DVA for children undergoing anesthesia. Patients with difficult intravenous access were more likely to have the attending anesthesiologist insert the successful IV and require ultrasound use. Although many of the factors associated with DVA in our study have been confirmed by others,^2^, ^3^, ^4^, ^5^, ^6^, ^7^, ^8^, ^9^, ^12^, ^14^ our study adds to the literature by showing a strong association of non‐Hispanic Black race/ethnicity with DVA, in addition to showing that patients with DVA were more likely to require the use of ultrasound and the attending anesthesiologist for successful IV placement. Early identification of patient risk factors for DVA can enable the anesthesiologist to preemptively modify their management to maximize chances for successful IV insertion. A prospective study addressing these potential interventions after identifying patients at high risk for DVA in the operating room is a future direction to explore.

In contrast to our findings that non‐Hispanic Black race/ethnicity was associated with DVA, Cuper and colleagues found that dark skin color did not have a significant effect on IV insertion success rates in pediatric patients in the OR.^2^ However, Yen and colleagues reported that darker skin shade was a predictor for pediatric DVA in the ED, while Galvez and colleagues reported that Black race was associated with pediatric DVA in the OR.^9^, ^12^ We were unable to determine the darkness of skin color due to the retrospective nature of our study and recognize that patients who identify as Black may have variation in skin color. It is unknown if the association of Black race with DVA is due to the darkness of skin color causing difficulty in seeing veins or to other factors associated with racial health disparities. However, in a post hoc multivariate logistic regression with ultrasound use as the dependent variable, non‐Hispanic Black patients were more likely to have ultrasound used for their IV insertion even after controlling for DVA status and demographic, comorbid, and hospital characteristics. This suggests that clinicians in our hospital recognized that patients identifying as Black, who likely have darker skin, may benefit from early ultrasound use. A prospective study looking at darkness of skin color versus racial/ethnicity category is warranted to test these differences.

Ultrasound use was strongly associated with DVA in our study. In our institution, ultrasound use is a limited resource, so preference is often given to patients with known or predicted difficult access. Since it is often used as a rescue method after patients have failed landmark techniques, it is not surprising that ultrasound is associated with DVA in this study. A randomized controlled trial by Vinograd and colleagues showed improved first attempt success with ultrasound compared to landmark techniques.^17^ Unfortunately, we are unable to determine the first attempt success rates in patients with known or suspected DVA when practitioners chose to use ultrasound as the initial technique due to the retrospective nature of this study. Interestingly, the practice of using ultrasound for IV insertion varies widely. In our study, ultrasound was used for 9.4% for patients with nondifficult IV access and 21.3% for patients with difficult IV access, whereas Galvez and colleagues reported an overall adjunct device (ultrasound and vein illumination devices) rate of less than 1% and O'Reilly‐Shah reported using ultrasound for 8.2% of IV insertions. The difference in ultrasound use may be due to differences in institutional culture or how the practice of pediatric anesthesia is changing over time given that the data from Galvez's study were from 2016 and the data from O'Reilly‐Shah's study were from 2019.

Our finding that attending anesthesiologists were more likely to insert the successful IV in patients with DVA warrants further discussion. Though there is not a formalized policy at the authors' institution, many attending anesthesiologists will take over attempts at IV insertion after another practitioner fails twice. We believe this institutional practice is the reason that attendings anesthesiologists were associated with DVA, but we cannot predict this with certainty because only the practitioner associated with the successful attempt was documented in the IV procedure note. However, in contrast, Cuper and colleagues found that attending anesthesiologists were less likely to have first attempt success than anesthetic nurses, postulating that the anesthetic nurses' high skill and long period of practical experience explained the difference.^2^

There were several limitations of this study. First, we were unable to determine causation due to the observational nature of this study. Second, this study took place at a tertiary care children's hospital, so it is unknown our findings are generalizable to other institutions. Third, the number of IV attempts was self‐reported in the medical record; therefore, it is possible that some patients were misclassified as having DVA. Third, we did not adjust for fasting status as a factor for difficult vascular access; however, Galvez did not find an association between fasting status and multiple IV insertions.^9^ Fourth, presence of patient comorbidities (prematurity, congenital heart disease, and renal disease) were based on ICD‐9/10‐CM billing codes associated with the patient's medical record so there is a chance of misclassification bias.^26^ Fifth, only the method used for the successful IV attempt is documented in our EMR. We were unable to determine whether ultrasound or landmark techniques were used for previous IV attempts. Finally, we were unable to determine whether inhalation induction was used before IV catheter insertion because IV insertion documentation occurs after induction and the timestamp of the IV procedure note is the only way we can determine IV insertion time. However, most anesthesiologists would agree that it is more demanding to cannulate a vein in an awake and moving child.

In conclusion, DVA in the operating room was associated with non‐Hispanic Black race, younger age, higher BMI, and higher ASA physical status. Patients who experienced DVA in the OR were more likely to have had ultrasound utilized and the attending anesthesiologist inserting the successful IV catheter. Further prospective studies are needed to determine if prediction rules can be developed to guide practice in the operating room.

## CONFLICT OF INTEREST

None.

## AUTHOR CONTRIBUTIONS

HB helped with the design of the study, analysis and interpretation of data, and drafting the manuscript. JH helped with the acquisition and analysis of data and revising the manuscript. EC helped with the interpretation of the data and revising the manuscript. MK helped with the conception and design of the study, interpretation of data, and revising the manuscript. JB helped with the analysis and interpretation of the data and revising the manuscript. All the authors above approved of the final version of the manuscript and are accountable for all aspects of this work.

## Data Availability

The data that support the findings of this study are available from Ann and Robert H. Lurie Children's Hospital of Chicago. Restrictions apply to the availability of these data, which were used under license for this study. Data are available from the corresponding author with the permission of Ann and Robert H. Lurie Children's Hospital of Chicago.
